# Buyang Huanwu Decoction promotes the neurological recovery of traumatic spinal cord injury via inhibiting apoptosis, inflammation, and oxidative stress

**DOI:** 10.1002/iid3.933

**Published:** 2023-07-12

**Authors:** Xu Li, Yingjun Song, Yang Yang, Guofu Zhang

**Affiliations:** ^1^ Department of Trauma Orthopedics Affiliated Hospital of Jiangxi University of Chinese Medicine Nanchang China

**Keywords:** BYHWD, MSCs, spinal cord injury, STAT, traditional Chinese medicine

## Abstract

**Background:**

The incidence rate of spinal cord injury (SCI) is increasing, and the mortality or disability rate caused by SCI remains high in the world. Buyang Huanwu Decoction (BYHWD) is a kind of Traditional Chinese medicine, and it is believed to be effective in several kinds of nervous system diseases. Whether BYHWD could improve SCI and the potential function mechanism remain unclear.

**Methods:**

SCI animal model was established by damaging T10 spinal cord. Animals experiments included five groups as follows: Sham, SCI, SCI+BYHWD, SCI+mesenchymal stromal cells (MSCs), and SCI+BYHWD+MSCs. H_2_O_2_‐treated cells (100 µM, 6 h) were used to simulate SCI damage in vitro, which included five groups as follows: control, H_2_O_2_, H_2_O_2_+BYHWD, H_2_O_2_+MSCs, and H_2_O_2_+BYHWD+MSCs. The behavioral function was evaluated with Tarlov and inclined plated test score. Western blot analysis and immunohistochemical staining were used to detect protein expression. The levels of superoxide dismutase (SOD), catalase (CAT), malondiadehyde (MDA), interleukin (IL)‐1β, tumor necrosis factor‐α, and IL‐6 in serum were measured with commercial enzyme‐linked immunosorbent assay kits. terminal deoxynucleotidyl transferase dUTP nick end labeling staining and flow cytometry were performed to measure apoptosis in vivo and in vitro levels. Gene expression profiling analysis was performed to analyze differential expression genes.

**Results:**

BYHWD suppressed apoptosis and accelerating cell proliferation after SCI. Recovery of neurofunction, inhibition of inflammatory response, and oxidative condition were achieved by BYHWD and MSCs. The expression levels of gp130/Janus kinase/signal transducers and activator of transcription (JAK/STAT) were suppressed by BYHWD and MSCs, both in vivo and in vitro. BYHWD and MSCs markedly promoted cells viability and inhibited apoptosis. Greater gene expression difference was observed between group control and H_2_O_2_ through gene expression profiling analysis. The recovery effects of traumatic SCI by BYHWD were similar to MSCs, and synergies effects were observed in several items.

**Conclusion:**

BYHWD could increase Tarlov score and Basso, Beatie, and Bresnahan functional score, inhibit apoptosis, inflammatory response, and oxidative condition after SCI. The expression level of gp130/JAK/STAT axis was suppressed by BYHWD. BYHWD might be a new therapeutic strategy for the prevention or treatment of SCI.

## Graphical abstract

BYHWD could promote the neurofunction recovery, inhibit apoptosis, inflammatory response, and oxidative condition after SCI. BYHWD might meliorate the recovery of SCI rats through inhibiting gp130/JAK/STAT. This study might provide a new therapeutic strategy for the prevention or treatment of SCI.

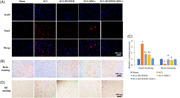

## 1 INTRODUCTION

The incidence rate of spinal cord injury (SCI) is increasing in the world. SCI can cause serious physical dysfunction and psychological disorder.^1^ The lack of treatment not only seriously affects the quality of life of patients, but also causes a great health burden to patients and their families.^2^ Traditional treatment mainly depends on drugs and surgery. Although it can alleviate the disease to a certain extent, the curative effect is limited and cannot fundamentally solve the problem of patients.^3^

SCI is mainly divided into primary injury and secondary injury. The primary injury is often irreversible, the current treatment is mainly depending on prevention and treatment of secondary SCI.^4^, ^5^ Traditional Chinese medicine has been believed to present potential effectiveness for the treatment of nervous system diseases including SCI. Buyang Huanwu Decoction (BYHWD) comprised seven medicinal herbs, including Raw Astragalus, Angelica, Earthworm, Red peony, *Ligusticum chuanxiong*, Peach kernel, and Safflower.^6^ The regulatory role of BYHWD in the central nervous system has been widely reported. BYHWD could meliorate intracerebral hemorrhage through target transfer RNA‐derived small RNA.^7^ BYHWD promoted neurogenesis via sirtuin1/autophagy pathway in a cerebral ischemia model.^8^ It was reported that BYHWD could attenuate intracerebral hemorrhage‐induced glial scar by inhibiting the expression of leukemia inhibitory factor in the rats.^9^ However, whether BYHWD could influence the improvement of SCI and the potential functioning mechanism have not been investigated.

Mesenchymal stromal cells (MSCs) have good differentiation ability and are easy to isolate and obtain. As seed cells, MSCs have achieved good results in the treatment of SCI.^10^ MSCs could migrate to the damaged site and differentiate into the damaged cells. In addition, MSCs can synthesize and secrete growth factors (vascular endothelial growth factor [VEGF] and glial‐derived nerve growth factor) to promote tissue repair.^11^, ^12^ In this study, we want to compare the effects of BYHWD on SCI with MSCs, and investigate whether BYHWD could exert a synergy effect with MSCs on SCI recovery.

Janus kinase/signal transducers and activator of transcription (JAK/STAT) signal transduction pathway is an important signal transduction pathway in cells, which is composed of JAK and STAT protein families, respectively.^13^ It was found that the contents of phosphorylated JAK2 and STAT3 in spinal cord tissue were increased after SCI and inhibition of JAK/STAT signaling pathway was closely related to nerve regeneration.^14^ However, if BYHWD could improve SCI through regulating JAK/STAT has not been investigated.

In this study, we established the SCI animal model, and investigate the influence of BYHWD, MSCs. We aimed to investigate the regulatory role of BYHWD in SCI, compared the function effect of BYHWD to MSCs, and explored the underlying mechanism preliminarily. We found that BYHWD might meliorate the function recovery of animals after SCI through inhibiting gp130/JAK/STAT signaling pathway. This study might provide a new thought for the prevention and treatment of SCI.

## 2 METHODS AND MATERIALS

### 2.1 Study medications

The herbal formula, BYHWD, comprised seven medicinal herbs including Raw Radix Astragalis, Chinese Angelica, Earthworm, Red peony root, Sichuan lovase rhizome, Peach seed, and Safflower (Table 1) based on Pharmacopoeia of the people's Republic of China. The BYHWD was obtained from the affiliated hospital of Jiangxi University of Chinese Medicine.

**Table 1:** Composition of BYHWD decoction.

Chinese name	Latin name	English name	Composition ratio (%)
Shenghuangqi	*Astragalus mongholicus Bunge*	Raw Radix Astragalis	50
Danggui	*Angelicae Sinensis Radix*	Chinese Angelica	8.3
Dilong	*Pheretima*	Earthworm	8.3
Chishao	*Paeoniae Radix Rubra*	Red peony root	8.3
Chuanxiong	*Chuanxiong Rhizoma*	Sichuan lovase rhizome	8.3
Taoren	*Persicae Semen*	Peach Seed	8.3
Honghua	*Carthami Flos*	Safflower	8.3

Abbreviation: BYHWD, Buyang Huanwu Decoction.

### 2.2 Cell culture and H_2_O_2_‐treated cell model

Rat spinal cord astrocytes purchased from Procell Life Science& Technology Co., Ltd (#CP‐R306) were used in this study. The cells were cultured with Dulbecco's modified Eagle's medium (Thermo) containing 10% fetal bovine serum (Thermo) in the incubator with 37°C and 5% CO_2_. After treatment with BYHWD (10 mg/mL) for 12 h, the cells were incubated with H_2_O_2_ (100 µM) for 6 h. Then, the cells were subjected to measurement of proliferation and apoptosis.

### 2.3 Establishment of animal model of SCI

All animals were used for experiments after 7 days of adaptive feeding. Sprague‐Dawley rats (female, 8–10 weeks) were used in this study. The SCI animal model was established as follows. After anesthesia, the rats were fixed on the operating table in prone position. A longitudinal incision with a length of 3 cm was made with T10 as the center to expose T10 spinal cord. The weight (10 g) fell freely from a height of 12.5 cm to T10 spinal cord, causing impact injury to T10 spinal cord. The complete paralysis of both lower limbs after operation proved that the modeling was successful. After operation, the animals were placed on the insulation pad until they woke up. In total, five groups (Sham, SCI, SCI+BYHWD, SCI+MSCs, and SCI+BYHWD+MSCs) were included in this research with eight rats in each group. The animals in the sham operation group only exposed T10 spinal cord without SCI. After the operation, the rats were assisted with artificial bladder compression every day and the rats resumed spontaneous micturition 2 weeks later. The animals in the group SCI+BYHWD was treated with intragastric BYHWD (25 g/kg) once a day for 28 days. The animals in the group Sham and SCI were treated with same amount of normal saline. The rats in the group SCI+MSCs were injected with MSCs (1 × 10^6^, 1 mL) twice a week for 4 weeks. The rats in the group SCI+BYHWD+MSCs were treated with BYHWD as described above and plus administration with MSCs (1 × 10^6^, 1 mL) twice a week for 4 weeks. After 4 weeks, animals were humanely killed at the end of the experimental period (isoflurane overdose while under anesthesia) and tissues were collected for experiment.

### 2.4 Behavior assessment

The modified Tarlov score was used for behavioral observation. The scoring rules are as follows: there is no autonomous movement, only nonreflective movement of hip and knee (0–1 points); movement of hip, knee, and ankle (2 points); can actively support weight and uncoordinated or occasionally coordinated gait (3 points); coordinated gait of forelimb and hindlimb, movement of interphalangeal joint between walking (4 points); and normal gait (5 points).

Inclined board test: on a rectangular board, put the rat's head forward, and the longitudinal axis of the body is perpendicular to the longitudinal axis of the inclined board, and gradually increase the angle between the board and the horizontal plane until the rat can maintain for just 5 s at the original position. This angle is the critical angle of the inclined plane. The critical angle of inclined plane of rats in each group was recorded.

The Basso, Beatie, and Bresnahan functional (BBB) score was performed in this study as described previously.^15^ The movement of trunk, tail, limbs, and hind of rats were recorded, and the BBB scores were evaluated.

### 2.5 Western blot analysis

The cells and tissues were lysed with protein was extracted. BCA kit was used to determine the protein concentration. Same amount protein samples were separated with 12% sodium dodecyl‐sulfate polyacrylamide gel electrophoresis. The protein samples were transferred to nitrocellulose membrane (Millipore) for 30–50 min. Primary antibodies were used to culture membranes overnight at 4°C. After washing with phosphate‐buffered saline (PBS) for three times, the membranes were incubated with second antibodies for 2 h at room temperature. After washing with PBS for three times, electrochemiluminescence exposure solution was added to the membranes. Quantity one software was used to analyze band gray. The antibodies used in this study were listed as follows: anti‐IL‐6 antibody (ab233706, Abcam), anti‐pAKT antibody (ab38449, Abcam), anti‐gp130 antibody (ab227058, Abcam), anti‐pJAK2 antibody (ab32101, Abcam), anti‐pSTAT3 antibody (ab267373, Abcam), anti‐GAPDH antibody (ab9485, Abcam), and goat anti‐Rabbit IgG (ab205718, Abcam).

### 2.6 Hematoxylin–eosin (HE) staining

Tissues were collected 4 weeks after SCI animal model induction. After killing, 2 cm injury site and the upper and lower spinal cord tissues were collected and embedded with paraffin. The spinal cord tissues were collected at the endpoint of animal experiment. After fixation with 4% paraformaldehyde (Beyotime) for 48 h, the tissues were embedded with paraffin. The tissues were cut into 10 μm sections. Then, HE were used to stain sections and an inverted optical microscope was used to observe the images.

### 2.7 Terminal deoxynucleotidyl transferase dUTP nick end labeling (TUNEL) staining

The sections were incubated with proteinase K (10 μg/mL, 10 min), hydrogen peroxide (0.3%, 10 min), and Triton X‐100 (0.1%, 10 min) successively. After washing with PBS, the sections were blocked with 5% goat serum (3 h). Then, the slides were incubated with 5‐bromo‐4chloro 3 indolyphosphate p toluidin salt/nitroblue tetrazolium chloride solution for 2 h. After washing with PBS, the slides were observed with a microscope (Carl Zeiss).

### 2.8 Measurement of superoxide dismutase (SOD), catalase (CAT), malondialdehyde (MDA), interleukin‐1β (IL‐1β), tumor necrosis factor‐α (TNF‐α), and IL‐6

The concentrations of SOD (Beyotime), CAT (Beyotime), MDA (Beyotime), IL‐1β (Nanjing Jiancheng), TNF‐α (Nanjing Jiancheng), and IL‐6 (Nanjing Jiancheng) in the serum were measured with commercial kits according to the instruction.

### 2.9 Apoptosis detection

After digestion with trypsin, the cells were centrifuged at 1500*g* for10 min. After removal of supernatant, the cells were suspended with PBS. The cells were cultured with propidium iodide and Annexin V–fluorescein isothiocyanate (Beyotime) in the dark for 20 min. Finally, flow cytometry was used to analyze cell apoptosis.

### 2.10 MTT (**3‐(4,5‐Dimethylthiazol‐2‐yl)‐2,5‐diphenyltetrazolium bromide**) assay

The proliferation of cells was detected by MTT assay. The cells were incubated with MTT regents for 4 h and the supernatant was removed. After adding 150 µL dimethyl sulfoxide, the optical density of at 450 nm was measured after 3 h.

### 2.11 Bromodeoxyuridine (Brdu) immunofluorescence staining

The cells were fixed with 4% paraformaldehyde for 15 min. PBS was used to wash cells three times. Cells were incubated with 0.5% Triton X‐100 at room temperature for 20 min. After washing with PBS for three times, 5% bovine serum albumin was used for blocking for 30 min. The cells were incubated with Brdu primary antibody (1:200, #5292, Cell Singnaling) for 4 h at room temperature. After washing with PBS for 3 times, second antibody was used to incubate cells for 45 min at room temperature. After washing with PBS for three times, 4′,6‐diamidino‐2‐phenylindole (#KGA215‐50, Keygen) was used to stain nucleus. Images were collected using a fluorescence microscope.

### 2.12 Gene expression profiling

Total RNA from each sample was quantified using the NanoDrop ND‐1000. The sample preparation was performed as below. Total RNA from each sample was amplified and transcribed into fluorescent cRNA. The labeled cRNAs were hybridized onto the Whole Mouse Genome Oligo Microarray (Agilent Technologies). After having washed the slides, the arrays were scanned by the Agilent Scanner G2505C. Agilent Feature Extraction software (version 11.0.1.1) was used to analyze acquired array images. Quantile normalization and subsequent data processing were performed using the GeneSpring GX v11.5.1 software package (Agilent Technologies). After quantile normalization of the raw data, genes were chosen for further data analysis. Differentially expressed genes were identified through fold‐change filtering.

### 2.13 Statistical analysis

SPSS 20.0 software was used for statistical analysis. All experiments were repeated at least three times and the quantitative results were expressed as mean ± SD. Independent sample *t* test was used for quantitative value comparison between the two groups, one‐way analysis of variance was used for quantitative analysis between multiple groups and student newman keuls method was used for pairwise comparison.

## 3 RESULTS

### 3.1 BYHWD improved histological injury and inhibited apoptosis after SCI

**Figure 1:**
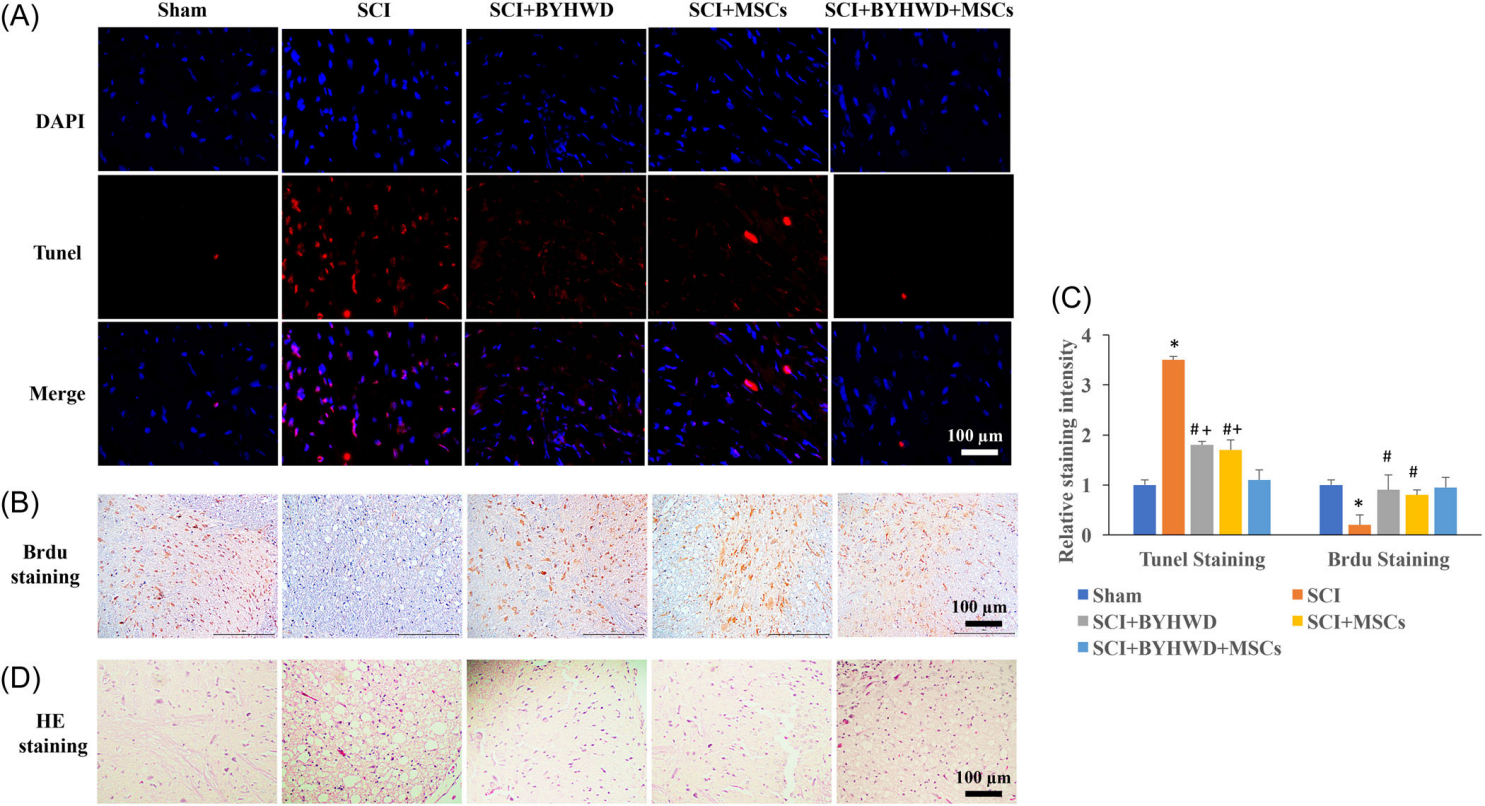
Buyang Huanwu Decoction (BYHWD) and inhibition of gp130/Janus kinase/signal transducers and activator of transcription (JAK/STAT) improved histological injury and inhibited apoptosis after spinal cord injury (SCI). (A) The apoptosis was evaluated with terminal deoxynucleotidyl transferase dUTP nick end labeling (TUNEL) staining. (B) The living cells in the spinal cord were detected with bromodeoxyuridine (Brdu) staining. (C) The cell apoptosis and living cells were analyzed. (D) Hematoxylin–eosin staining was performed to investigate the tissue injury. *p < .05 compared with the group sham. #*p* < .05 compared with the group SCI. +*p* < .05 compared with group SCI+BYHWD+mesenchymal stromal cells (MSCs).

In this study, TUNEL and Brdu staining were used to investigate cell apoptosis and living cells, respectively. We found that apoptosis was promoted and living cells were significantly decreased in the group SCI compared with group Sham (Figure 1A–C). However, treatment with BYHWD or MSCs remarkably reversed these trends. Meanwhile, simultaneous treatment with MSCs and BYHWD markedly suppressed cell apoptosis compared with group SCI+BYHWD or group SCI+MSCs (Figure 1A–C).

### 3.2 BYHWD improved neurofunction recovery, inhibiting inflammatory factors and oxidative stress after SCI

**Figure 2:**
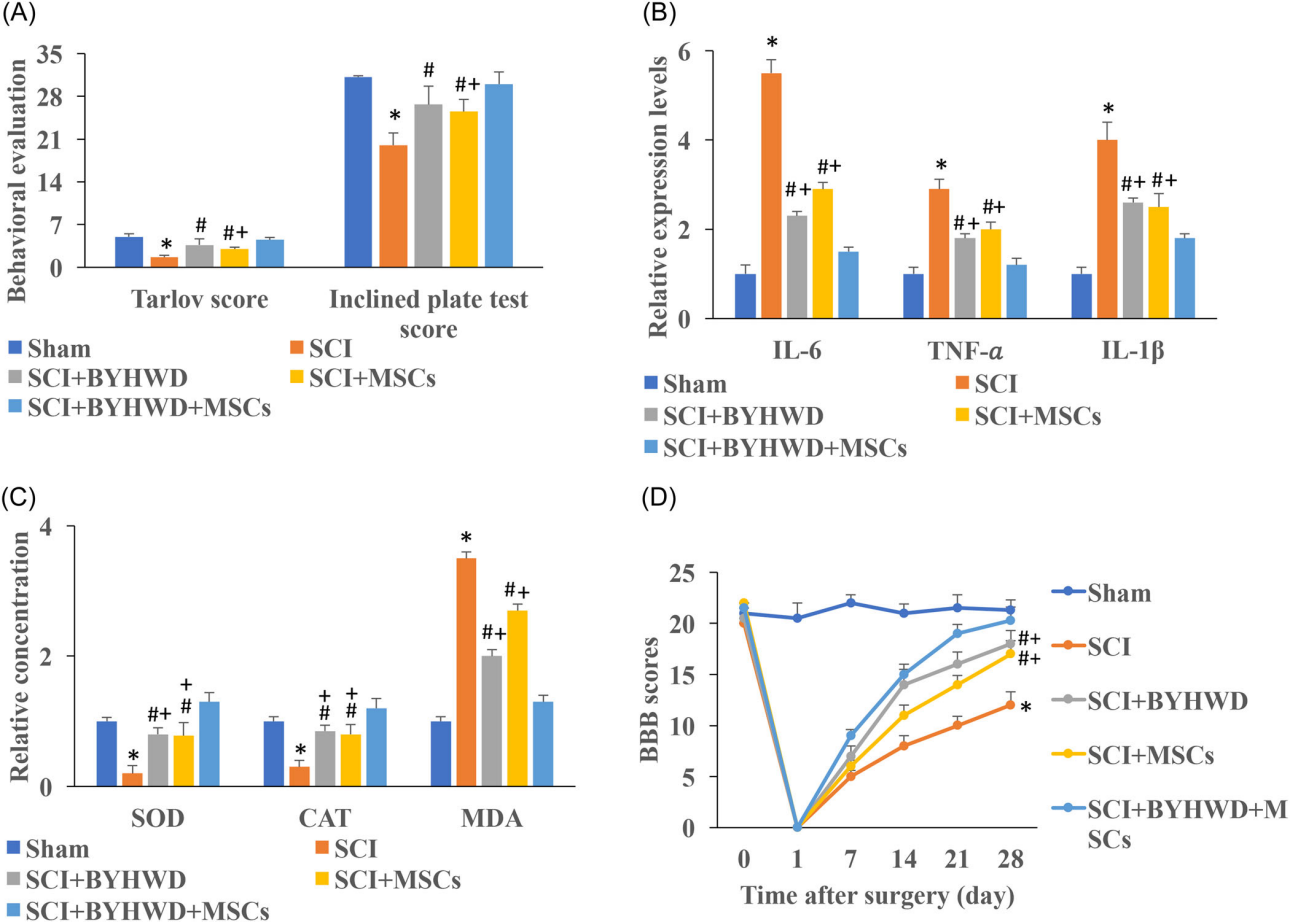
Buyang Huanwu Decoction (BYHWD) and inhibition of gp130/Janus kinase/signal transducers and activator of transcription (JAK/STAT) improved neurofunction recovery after spinal cord injury (SCI). (A) Tarlov and inclined plated tests were used to investigate neurofunction recovery. (B) The levels of interleukin‐1β (IL‐1β), tumor necrosis factor‐α (TNF‐α), and IL‐6 in the serum were detected. (C) The levels of MDA, SOD, and CAT in the serum were measured. (D) The Basso, Beatie, and Bresnahan functional (BBB) scores were performed to investigate neurofunction recovery. *p < .05 compared with the group sham; #*p* < .05 compared with the group SCI. +*p* < .05 compared with the group SCI+BYHWD+mesenchymal stromal cells (MSCs).

The behavioral function was evaluated with Tarlov and inclined plated test score system. After SCI, both Tarlov and inclined plated test scores were decreased. However, BYHWD and MSCs treatment significantly promoted these two items. In addition, simultaneous treatment with MSCs presented higher score compared with group SCI+MSCs (Figure 2A). To investigate the influence of BYHWD on the inflammatory factors' expression and oxidative stress response, the levels of IL‐1β, TNF‐α, IL‐6, SOD, CAT, and MDA were detected. BYHWD and MSCs could greatly suppress the levels of IL‐1β, TNF‐α, and IL‐6 compared with group SCI (Figure 2B). In addition, simultaneous treatment with MSCs and BYHWD presented stronger inflammation inhibition effect (Figure 2B). Meanwhile, the levels of inflammatory factors and oxidative state were evaluated. The increased MDA, decreased SOD and CAT induced by SCI were reversed by BYHWD or MSCs (Figure 2C). In addition, simultaneous treatment with MSCs and BYHWD treatment remarkably promoted SOD and CAT, but inhibited MDA compared with group SCI+BYHWD or group SCI+MSCs (Figure 2C). BBB scores were also performed to investigate the influence of MSCs and BYHWD on neurofunction recovery. Similar results to Tarlov and Inclined plated tests were obtained (Figure 2D). Single treatment with BYHWD or MSCs could markedly improve BBB scores compared with group SCI and simultaneous treatment with MSCs and BYHWD achieved a higher BBB score.

### 3.3 BYHWD significantly inhibited the expression of gp130/JAK/STAT signaling pathway in vivo

**Figure 3:**
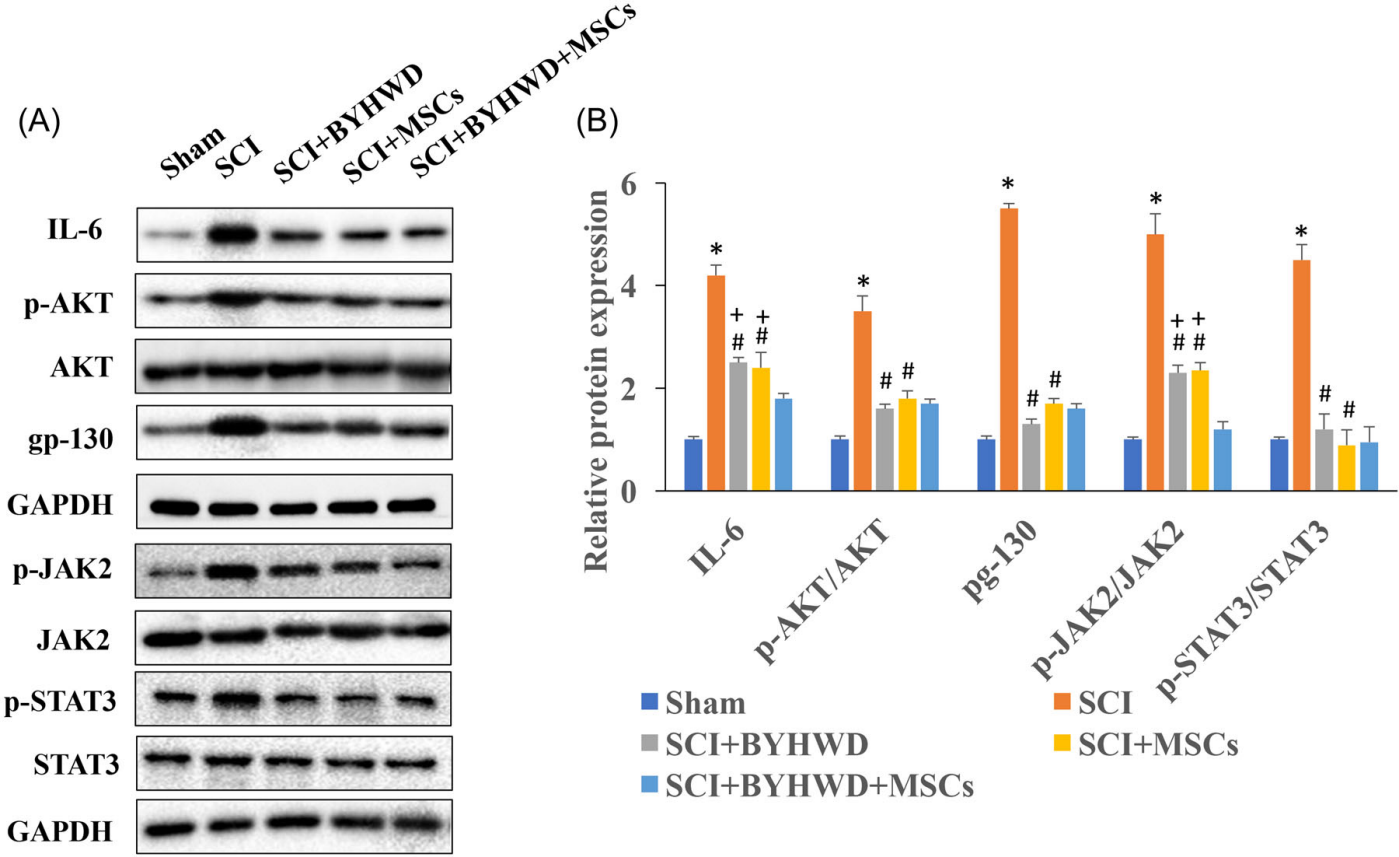
Buyang Huanwu Decoction (BYHWD) significantly inhibited the expression of gp130/Janus kinase/signal transducers and activator of transcription (JAK/STAT) signaling pathway in vivo. (A) The protein levels of interleukin (IL)‐6, p‐AKT/AKT, gp‐130, p‐JAK2/JAK2, and p‐STAT3/STAT3 in the spinal cord were measured with western blot analysis (*n* = 3). (B) The protein levels of IL‐6, p‐AKT/AKT, gp‐130, p‐JAK2/JAK2, and p‐STAT3/STAT3 were analyzed. *p < .05 compared with the group sham. #*p* < .05 compared with the group SCI. +*p* < .05 compared with the group SCI+BYHWD+mesenchymal stromal cells (MSCs).

gp130/JAK/STAT signaling pathway has been believed to be closely linked with the regulation of SCI and some gp130/JAK/STAT signaling pathway related proteins levels were measured. The protein expression levels of IL‐6, p‐AKT/AKT, gp‐130, p‐JAK2/JAK2, and p‐STAT3/STAT3 were greatly increased after SCI (Figure 3A,B). However, after treatment with BYHWD or MSCs, the levels of IL‐6, p‐AKT/AKT, gp‐130, p‐JAK2/JAK2, and p‐STAT3/STAT3 were markedly decreased compared with group SCI. In addition, simultaneous treatment with MSCs and BYHWD shown even stronger suppression effect in terms of the expression of IL‐6 and p‐JAK2/JAK2 compared with group SCI+BYHWD or group SCI+MSCs (Figure 3A,B).

### 3.4 BYHWD and MSCs markedly promoted cells viability and inhibited apoptosis in vitro

**Figure 4:**
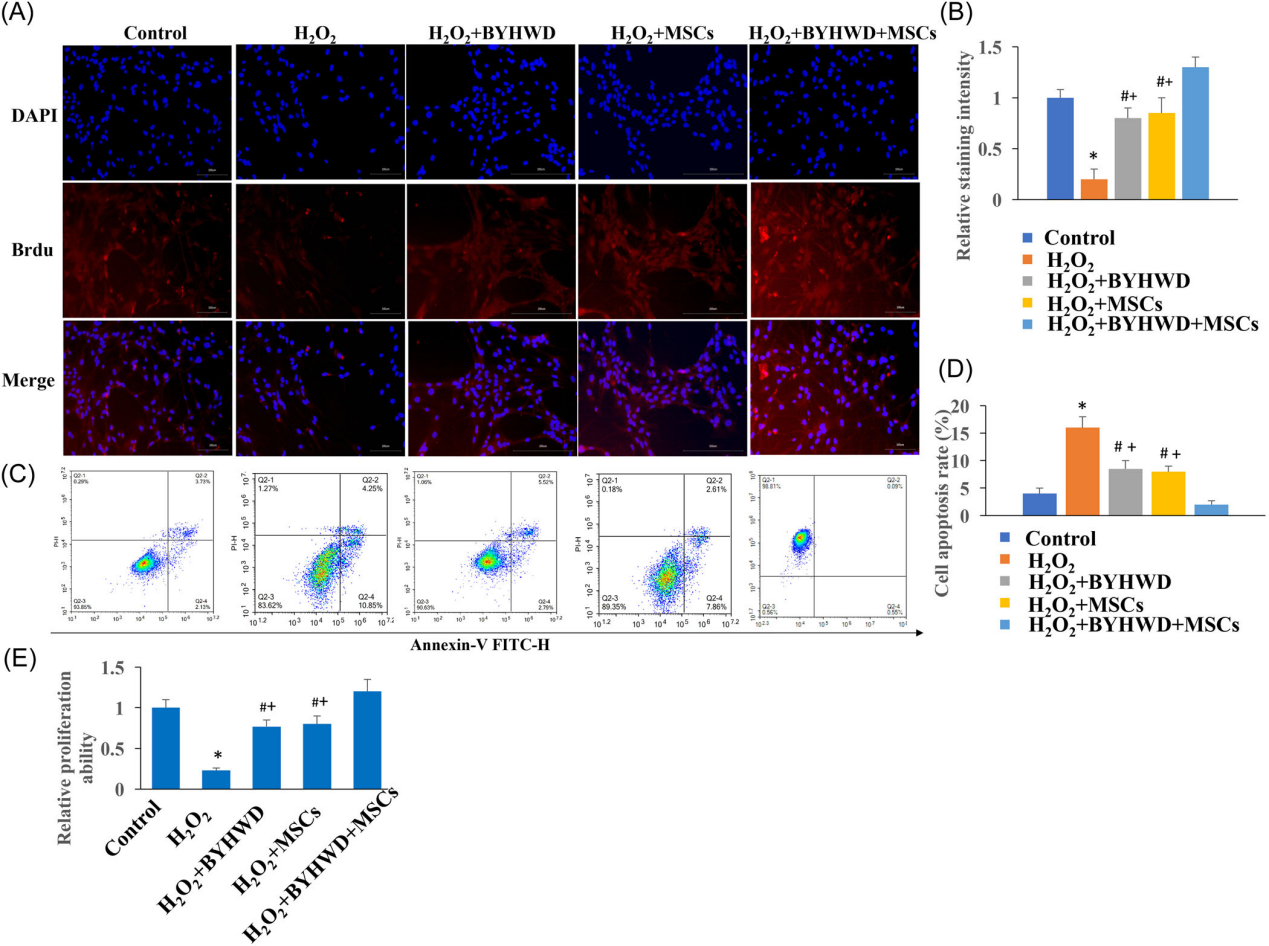
Buyang Huanwu Decoction (BYHWD) and mesenchymal stromal cells (MSCs) markedly promoted cells viability and inhibited apoptosis. (A) The cell proliferation was measured with bromodeoxyuridine (Brdu) staining. (B) The cell proliferation was analyzed. (C) The cell apoptosis was measured with flow cytometry. (D) The cell apoptosis was analyzed. *p < .05 compared with the group control. #*p* < .05 compared with the group H_2_O_2_. +*p* < .05 compared with the group SCI+BYHWD+MSCs.

H_2_O_2_‐treated astrocytes was used to imitate SCI in vitro. We found that the cell proliferation was inhibited and cell apoptosis was promoted after H_2_O_2_ induction (Figure 4A–D). However, BYHWD and MSCs treatment remarkably reversed the effect of H_2_O_2_. In addition, co‐treatment with MSCs significantly promoted cell viability, but suppressed cell apoptosis compared with group H_2_O_2_+BYHWD (Figure 4A–D). In addition, the influence of BYHWD and MSCs on astrocytes proliferation was performed with MTT assay (Figure 4E). The suppressed cell proliferation ability by H_2_O_2_ was remarkably promoted by BYHWD and MSCs, and simultaneous treatment with MSCs and BYHWD presented a stronger cell proliferation stimulation ability (Figure 4E).

### 3.5 BYHWD significantly inhibited the expression of gp130/JAK/STAT signaling pathway in vitro

**Figure 5:**
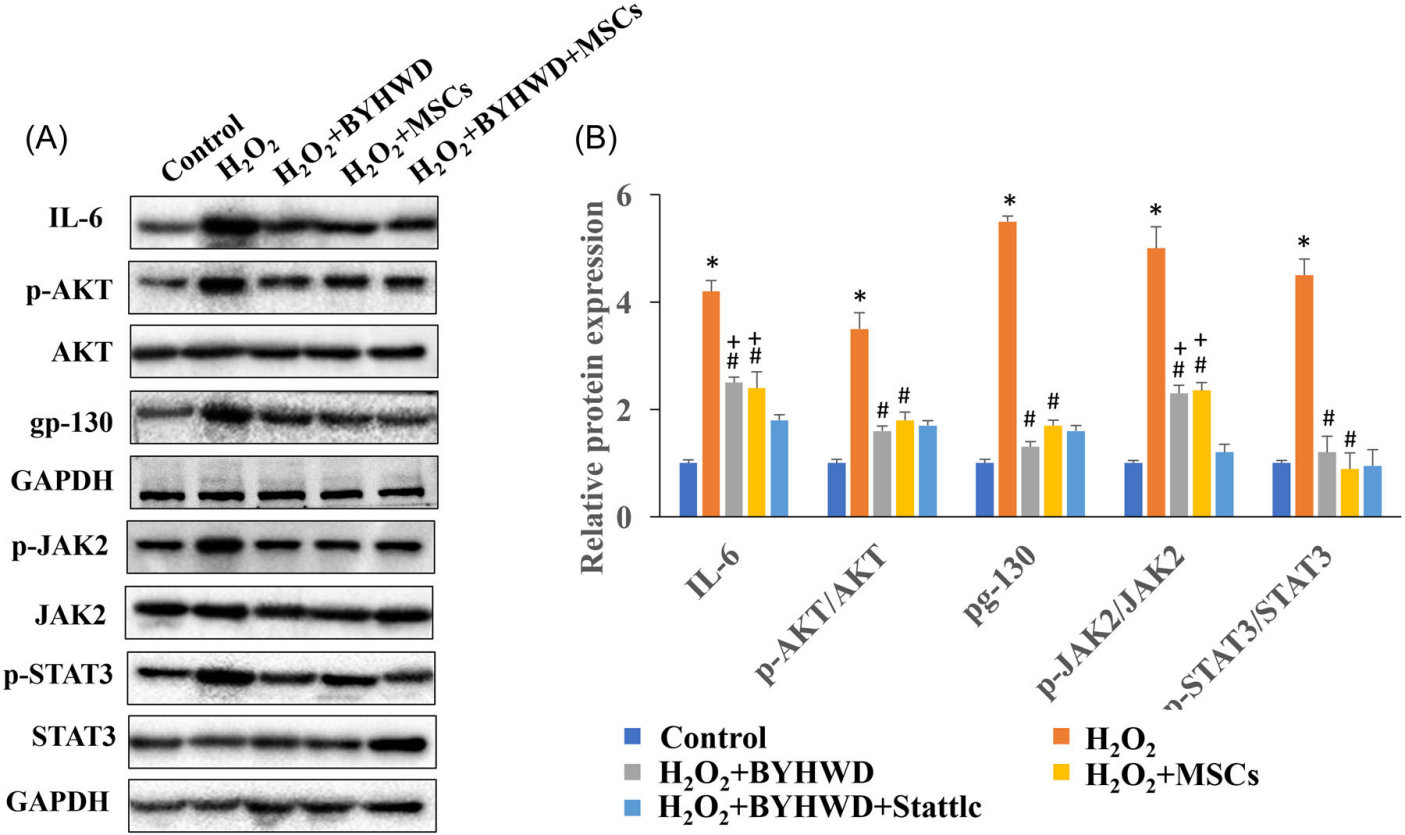
Buyang Huanwu Decoction (BYHWD) significantly inhibited the expression of gp130/Janus kinase/signal transducers and activator of transcription (JAK/STAT) signaling pathway in vitro. (A) The protein levels of interleukin (IL)‐6, p‐AKT/AKT, gp‐130, p‐JAK2/JAK2, and p‐STAT3/STAT3 were measured with western blot analysis (*n* = 3). (B) The protein levels of IL‐6, p‐AKT/AKT, gp‐130, p‐JAK2/JAK2, and p‐STAT3/STAT3 were analyzed. *p < .05 compared with the group control. #*p* < .05 compared with the group H_2_O_2_. +*p* < .05 compared with the group SCI+BYHWD+mesenchymal stromal cells (MSCs).

The influence of BYHWD on gp130/JAK/STAT signaling pathway was also validated in vitro. The protein levels of IL‐6, p‐AKT/AKT, gp‐130, p‐JAK2/JAK2, and p‐STAT3/STAT3 were significantly promoted after H_2_O_2_ induction (Figure 5A,B). However, BYHWD or MSCs treatment markedly decreased the levels of IL‐6, p‐AKT/AKT, gp‐130, p‐JAK2/JAK2, and p‐STAT3/STAT3. In addition, simultaneous treatment with MSCs and BYHWD shown even stronger inhibition effect on the expression of IL‐6 and p‐JAK2/JAK2 (Figure 5A,B).

### 3.6 Gene expression profiling analysis

**Figure 6:**
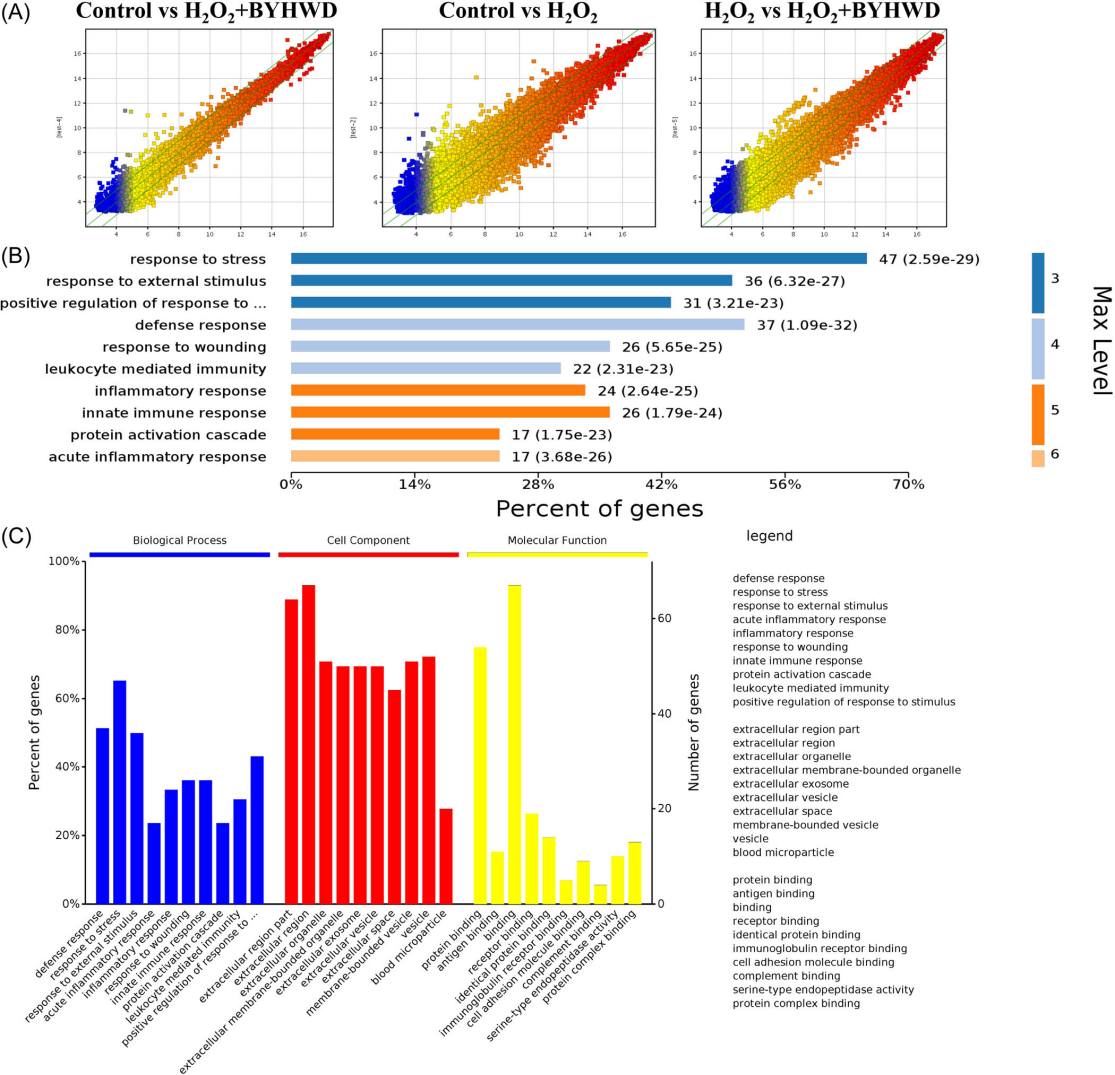
Buyang Huanwu Decoction (BYHWD) significantly inhibited the expression of gp130/Janus kinase/signal transducers and activator of transcription (JAK/STAT) signaling pathway in vitro. (A) Gene expression profiling was performed. (B) The differential expression genes between group H_2_O_2_ and H_2_O_2_+BYHWD were further analyzed. (C) Genes expression analysis related with biological process, cell component, molecular function was also performed.

We further perform gene expression profiling to analyze differential genes. The default fold‐change value of the three green broken lines is 2 (Figure 6A). The point above and below the green is the gene fold‐change ≥2.0 of the two groups. Larger green line dispersion indicates greater gene expression difference. We could observe that greater gene expression difference was observed between group control and H_2_O_2_ (Figure 6A). However, little differences in gene expression between group control and H_2_O_2_+BYHWD, between group H_2_O_2_ and H_2_O_2_+BYHWD were observed. In addition, the differential expression genes between group H_2_O_2_ and H_2_O_2_+BYHWD were further analyzed. Genes related with response to stress and external stimulus factors might be influenced by BYHWD during reversing the effects of H_2_O_2_ on cells (Figure 6B). In addition, genes expression analysis related with biological process, cell component, molecular function was also performed (Figure 6C).

## 4 DISCUSSION

Studies have shown that the neurological damage of SCI is caused by two mechanisms: primary injury (Mechanical damage and bleeding) and secondary injury (The toxic effects of edema, inflammatory reaction, ischemia, cytokines, and reperfusion on the spinal cord).^14^ For a long time, it has been believed that nerve cells cannot be regenerated after central nerve injury such as spinal cord. However, in recent years, a large number of basic experimental research and clinical practice have proved that the spinal cord has a certain ability of regeneration and repair, and secondary SCI can be remedied and reversed by intervention measures.^16^, ^17^ Therefore, it is necessary to explore novel therapeutic agents to meliorate SCI.

In the present study, traumatic SCI animal model was established through damaging T10 with weight (10 g) from a height of 12.5 cm, which is a common SCI study animal model. Complete paralysis of both lower limbs suggested the successful establishment of animal model. We found that significant higher cell apoptosis, inflammation response, oxidative stress condition, and lower living cells were observed in the established SCI animal model. The regulatory role of BYHWD in the central nervous system has been identified. It was reported that BYHWD could significantly reduce cerebral ischemia/reperfusion damage via suppressing metabotropic glutamate receptor‐1 RNA and glutamate release.^18^ BYHWD inhibited cerebral ischemia/reperfusion injury by promoting angiogenesis through SIRT1/VEGF pathway.^19^ In addition, BYHWD combined with BMSCs transplantation promoted recovery after SCI through rescuing axotomized red nucleus neurons.^20^ However, the specific function mechanism and molecule targets remain unknown.

Neuronal apoptosis is one of the causes of neurological dysfunction after SCI, and JAK/STAT is proved to be closely linked with the regulation of cell apoptosis. Several reports suggested that JAK/STAT signaling pathway was closely involved in the regulation of SCI. For example, microRNA‐125b could accelerate the regeneration and repair of SCI by targeting JAK/STAT pathway.^13^ In addition, the improvement of motor function recovery and reduce of spinal cord edema after SCI were achieving by Curcumin through inhibiting JAK/STAT signaling pathway.^21^ Therefore, we suspected that if BYHWD could play a regulatory role after SCI. In this study, we demonstrated that the gp130/JAK/STAT signaling pathway was significantly inhibited by BYHWD after SCI. In addition, supplementary treatment with MSCs markedly promoted the recovery induced by BYHWD after SCI. There data indicate that BYHWD might exert protective effect through inhibiting gp130/JAK/STAT signaling pathway. It was reported that BYHWD could improve neural recovery after SCI in rats through increasing mammalian target of rapamycin (mTOR) signaling pathway and autophagy.^22^ However, overexpression of mTOR and JAK‐STAT in neurons has several harmful consequences, including increased levels of reactive oxygen species and neuronal apoptosis, which suggested the complicated role of mTOR in SCI.^23^ Further study needs to be performed to validate the regulatory function of mTOR. In addition, BYHWD combined with MSCs transplantation promotes recovery after SCI by rescuing axotomized red nucleus neurons.^20^ We found that BYHWD combined with MSCs presented remarkable recovery of neurological function after SCI, but the combination treatments didn't show significant influence on the expression of gp130/JAK/STAT signaling pathway compared to signal treatment with BYHWD or MSCs. The underlying mechanism of synergistic effect of BYHWD and MSCs needs to be explored. Previous studies indicated that SCI was involved in inflammation or neuroinflammatory, which is regulated by nuclear factor‐κB (NF‐κB) and mitogen‐activated protein kinase (MAPK) signaling pathways.^24^, ^25^ If BYHWD could regulate SCI through affecting NF‐κB and MAPK signaling pathways is an interesting research direction. In addition, SCI is correlated with angiogenesis and scar formation in the local microenvironment of SCI.^26^ Therefore, the validation of pro‐angiogenesis effects in SCI by BYHWD might unfold the regulatory mechanism.

After traumatic injuries of the central nervous system, including SCI, astrocytes surrounding the lesion become reactive and typically undergo hypertrophy and process extension.^27^ Astrocytes are closely linked with spinal cord tissue repair process, and the role of astrocytes is complicated. Astrocyte proliferation is important for tissue repair and function recovery^27^ in the early stage after SCI. However, the glial scarring caused by astrocyte in the late stage after SCI could further inhibit axonal regeneration. This is the contradictory role of astrocyte in the repair process after SCI. The role of astrocyte in the repair process of SCI needs to be further explored at different stages of SCI. Therefore, we want to investigate the potential regulatory role of BYHWD in astrocytes.

Several reports indicate that MSCs might be the potential effective therapy for the SCI. MSCs can promote the repair of SCI by stimulating the growth of spinal cord nerve, guiding the regeneration of injured nerve, promoting growth factors secretion for nutrition supplement, increasing neovascularization, inhibiting inflammation, and reducing oxidative stress.^28^, ^29^, ^30^, ^31^ We demonstrated that MSCs accelerated the recovery of animals after SCI, and the gp130/JAK/STAT signaling pathway was also suppressed by MSCs. If BYHWD could play a synergy effect with MSCs on the recovery of SCI needs to be further investigated.

## 5 CONCLUSIONS

In summary, we found that BYHWD could increase Tarlov and inclined plate test scores after SCI. In addition, significant suppression of apoptosis, inflammatory response, and oxidative condition were achieved by BYHWD. We suspected that BYHWD might meliorate the recovery of SCI rats through inhibiting gp130/JAK/STAT. This study might provide a new insight for the treatment and prevention of SCI through gp130/JAK/STAT.

## AUTHOR CONTRIBUTIONS

**Xu Li**: Project administration; software; validation. **Yingjun Song**: Data curation; methodology; project administration. **Yang Yang**: Project administration; software; validation. **Guofu Zhang**: Conceptualization; funding acquisition; writing—original draft; writing—review & editing.

## CONFLICT OF INTEREST STATEMENT

The authors declare that they have no conflict of interest.

## ETHICS STATEMENT

The study has been approved by the Ethics Committee of Affiliated Hospital of Jiangxi University of Chinese Medicine.

## Data Availability

Data supporting this study has been presented in the manuscript, the data required by editor, reviewer and reader could be provided by the corresponding author.
